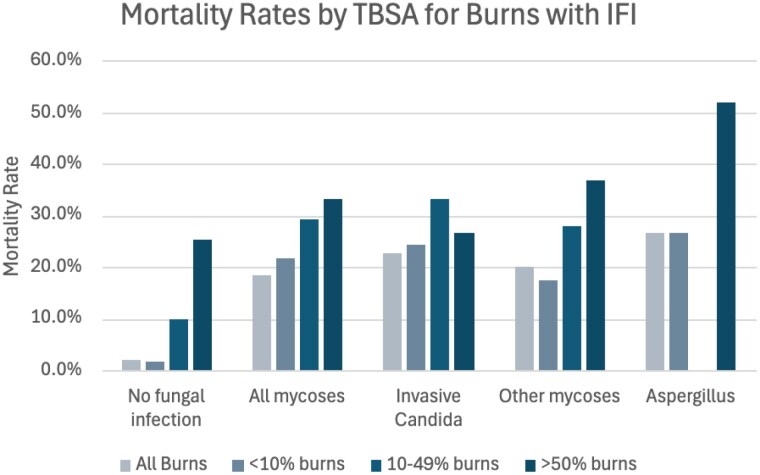# 582 Invasive Fungal Infection Increases Mortality Risk After Burn Injury

**DOI:** 10.1093/jbcr/iraf019.211

**Published:** 2025-04-01

**Authors:** Allison Frederick, Savannah Skidmore, Steven Kahn, Rohit Mittal

**Affiliations:** Medical University of South Carolina; Medical University of South Carolina; Medical University of South Carolina; Medical University of South Carolina

## Abstract

**Introduction:**

While invasive fungal infections (IFI) can be deadly for any hospitalized patient, a high-risk subset are those with burn injuries due to their inherently immunocompromised state. There is a paucity of literature on IFI in this population, making it difficult to evaluate the true risk. This novel study captures data from across the US using a commercially available multi-institutional dataset derived from electronic health record data and is the largest study to date evaluating mortality associated with IFI in burn patients.

**Methods:**

Inclusion criteria identified all patients with an ICD diagnosis of a burn from 2002-2024; IFI was defined as an ICD diagnosis of fungal mycoses with systemic antifungal treatment. This cohort was matched for gender, age and TBSA and compared to a control cohort of burn patients without mycoses or antifungal treatment. TBSA was further divided into three subcategories of < 10%, 10-49% and >50% TBSA burns to analyze differences in mortality.

**Results:**

The IFI cohort contained 3,326 patients while the control cohort was comprised of 706,463 patients. After matching, the risk of mortality for all IFI in burn patients was 18.5% compared to 1.9% in controls (RR: 9.8, 95% CI 7.2-13.2; p <.0001). Additionally, when stratified by TBSA there was a stepwise increase in mortality with TBSA: 21.6% for < 10% TBSA (RR: 12.4, 95% CI 6.6-23.4; p <.0001), 29.0% for 10-49% TBSA (RR: 2.7, 95% CI 1.8-4.2; p <.0001) and 33.1% for >50% TBSA (RR: 1.3, 95% CI 0.9-1.9; p=.02). Stratifying by organism, the 3 most common and deadly IFI among all burn patients were aspergillus, invasive candida and other mycoses with mortality rates of 26.7%, 22.7%, 20.0% respectively. Notably, aspergillus had the highest mortality rate of 26.7% (RR: 6.6, 95% CI 3.5-12.5; p <.0001) and when stratified by TBSA it substantially increased: 21.6% for < 10% TBSA (RR: 1.9, 95% CI 0.9-3.8; p <.06) and 51.9% for >50% TBSA (RR: 1.4, 95% CI 0.8-2.6; p=.3).

**Conclusions:**

In all burns, IFI is associated with a nearly 10-fold increase in mortality. An incremental increase is noted when stratified by TBSA and surprisingly, even small burns with IFI have an over 20% risk of mortality. All IFI are high risk, with survival differences based on organism, but aspergillus infection is associated with mortality in over ¼ of infected patients. When using a large deidentified database, associations may be drawn, but we cannot conclude that mortality was caused solely by IFI. Additionally, the lack of specificity in ICD-coding for fungal infections is problematic as large cohorts (other mycoses and unspecified mycoses) could be representing a wide range of fungal infections and we cannot identify which organisms are represented (i.e. curvularia, mucor, fusarium, etc.).

**Applicability of Research to Practice:**

IFI in burn patients present a challenging and often deadly problem; as the largest and most comprehensive study of IFI in burns, this study supports the early detection and consideration of antifungal prophylaxis in burns.

**Funding for the Study:**

N/A